# Worksite interventions for preventing physical deterioration among employees in job-groups with high physical work demands: Background, design and conceptual model of FINALE

**DOI:** 10.1186/1471-2458-10-120

**Published:** 2010-03-09

**Authors:** Andreas Holtermann, Marie B Jørgensen, Bibi Gram, Jeanette R Christensen, Anne Faber, Kristian Overgaard, John Ektor-Andersen, Ole S Mortensen, Gisela Sjøgaard, Karen Søgaard

**Affiliations:** 1 National Research Centre for the Working Environment, Copenhagen, Denmark; 2 Institute of Sports Science and Clinical Biomechanics, University of Southern Denmark, Odense, Denmark; 3 Department of Sport Science, Aarhus University, Denmark; 4 Multidisciplinary Pain Clinic, Primary Care Region Skåne, Malmö, Sweden; 5 Dept. of Occupational and Environmental Medicine, Bispebjerg University Hospital, Copenhagen, Denmark

## Abstract

**Background:**

A mismatch between individual physical capacities and physical work demands enhance the risk for musculoskeletal disorders, poor work ability and sickness absence, termed physical deterioration. However, effective intervention strategies for preventing physical deterioration in job groups with high physical demands remains to be established. This paper describes the background, design and conceptual model of the FINALE programme, a framework for health promoting interventions at 4 Danish job groups (i.e. cleaners, health-care workers, construction workers and industrial workers) characterized by high physical work demands, musculoskeletal disorders, poor work ability and sickness absence.

**Methods/Design:**

A novel approach of the FINALE programme is that the interventions, i.e. 3 randomized controlled trials (RCT) and 1 exploratory case-control study are tailored to the physical work demands, physical capacities and health profile of workers in each job-group. The RCT among cleaners, characterized by repetitive work tasks and musculoskeletal disorders, aims at making the cleaners less susceptible to musculoskeletal disorders by physical coordination training or cognitive behavioral theory based training (CBTr). Because health-care workers are reported to have high prevalence of overweight and heavy lifts, the aim of the RCT is long-term weight-loss by combined physical exercise training, CBTr and diet. Construction work, characterized by heavy lifting, pushing and pulling, the RCT aims at improving physical capacity and promoting musculoskeletal and cardiovascular health. At the industrial work-place characterized by repetitive work tasks, the intervention aims at reducing physical exertion and musculoskeletal disorders by combined physical exercise training, CBTr and participatory ergonomics. The overall aim of the FINALE programme is to improve the safety margin between individual resources (i.e. physical capacities, and cognitive and behavioral skills) and physical work demands, and thereby reduce the physical deterioration in a long term perspective by interventions tailored for each respective job-group.

**Discussion:**

The FINALE programme has the potential to provide evidence-based knowledge of significant importance for public health policy and health promotion strategies for employees at high risk for physical deterioration.

**Trial registrations:**

ISRCTN96241850, NCT01015716 and NCT01007669

## Background

Work ability reflects the relation between the capacity and demands of the worker [1]. When the workers capacity does not exceed the demands at work with a certain safety margin, this may be expressed as a decreased work ability [2]. The importance of good work ability is highlighted by the relation between lowered work ability and stress and burnout [3], chronic diseases [4], long term sickness absence [5-7], early retirement from the labour market [8,9] and all cause mortality [10]. Accordingly, a one point reduction in work ability on a single item 10 point scale is recently shown to increase the risk for long term sickness absence by 15%, and early retirement from the labour marked by 33% [7].

Workers with high physical work demands are well documented to be at elevated risk for impaired work ability [11-13], musculoskeletal disorders [14], cardiovascular disease [15], all-cause mortality [16], long term sickness absence [17] and early retirement from the labour market [7]. Specifically, prolonged standing, highly repetitive work, heavy lifting, working with the hands lifted to shoulder height or higher, and working with the back twisted or bent forward are physical exposures, that have been shown to predict impaired work ability, musculoskeletal disorders and enhance long term sickness absence [14,17,18]. Therefore, workers in job groups exposed to these physical factors at work are at particular need for health promoting initiatives for preserving or improving their work ability [11].

Previous initiatives applying individual counselling and education among employees with high physical work demands have not been able to show positive effects for improving work ability [19]. In contrast, physical exercise training has been shown to prevent impairment of work ability [20]. Because high physical work demands does not have the same positive effect on physical capacity as physical exercise training [21-25], it may be effective to improve physical capacity and preserve work ability among employees with high physical work demands through physical exercise training. Accordingly, physical exercise training interventions have shown positive effects on work ability [11,26]. The international recommendation of health promoting physical exercise training for healthy adults is at least 30 min moderate physical activity 5 days per week [27]. However, among employees with high physical work demands, specific health promoting physical exercise training recommendations (i.e. type, frequency, duration and intensity) remain to be established. Another initiative for preserving work ability among employees with high physical work demands is to reduce the relative workload by either participatory ergonomic intervention [28] or by reducing the excessive body weight [29]. By improving working techniques and cooperation with colleagues, the physical overload and peak loads can be reduced [28]. Reduction in excessive body weight may lower the relative workload on both the musculoskeletal and cardiovascular system, and therefore preserve work ability [29,30]. A third initiative for preserving work ability among employees with high physical work demands may be to improve their ability to cope with their musculoskeletal disorders by cognitive behavioral theory based training (CBTr). Cognitive behavioral therapy interventions are previously shown effective for facilitation of early return to work [31-33]. However, the effects of these initiatives for primary prevention of reduced work ability and sickness absence among employees with high physical work demands still remain to be established.

In the last decade, more focus has been on the workplace as a convenient arena for health promoting initiatives [26] such as smoking cessation [34], promotion of physical activity [35], dietary intake modification [36] reduction of overweight [37], reduction of alcohol consumption [38], prevention of musculoskeletal disorders [39] and prevention of sickness absence among employees with musculoskeletal disorders [40]. However, RCTs targeting the physical workload, lifestyle factors (e.g. physical exercise training) and physical capacity and pain-related cognitive and behavioral skills for preserving sufficient levels of work ability are still lacking [11].

In the FINALE programme, health impairing effects originating from a mismatch between individual capacities and physical work demands (i.e. musculoskeletal disorders, poor work ability and sickness absence) is defined as physical deterioration. The overall aim of the FINALE programme is to evaluate effects on 1) the balance between individual capacities (i.e. ergonomic skills, muscular strength, aerobic capacity, postural stability and pain-related cognitive and behavioral skills) and physical work demands (i.e. physical exertion and reduced excessive body weight), and 2) the resulting effects on physical deterioration (i.e. musculoskeletal disorders, work ability and sickness absence) from tailored interventions to the individual capacities, physical work demands and health profile of employees from 4 job groups (i.e. cleaners, health-care workers, construction workers and industrial workers). The consequent main outcomes are musculoskeletal disorders, work ability, physical capacity, body mass index (BMI), kinesiophobia, rate of physical exertion during work and sickness absence. The primary outcome is specifically tailored to each respective intervention.

## Methods and design

### Study design

The FINALE programme consists of 3 randomized controlled trials (RCT) and 1 exploratory case-control study involving several workplaces.

### Study population

Table 1 illustrates the predominant sex, physical work characteristics, physical capacities and health profile based on previous studies of the four study populations of the FINALE programme. Moreover, the intervention applied to each of the study populations is reported. Two of the job groups (i.e. health care workers and cleaners) are predominantly female employees, and two job groups (industrial workers and construction workers) are predominantly male employees. All job groups are characterized by a large amount of standing and walking. The industrial workers and cleaners are exposed to a large extent of repetitive work with moderate force demanding tasks [41,42]. The health care workers and construction workers are exposed to heavy lifting and other demanding tasks with high peak forces [12,13,25,43,44]. Previous studies show that all job groups are characterized by low individual physical capacities [12,23,25,41,45,46], high prevalence of musculoskeletal disorders, sickness absence and disability pension [41,43,47-53]. In particular, health care workers are characterised by having a relatively high prevalence of overweight [12], and construction workers for having a number of risk factors for cardiovascular diseases, e.g. high BMI [9,13,18].

**Table 1:** The study populations, predominant sex, physical work demands, individual capacities and health profile based on previous studies, and interventions comprising the FINALE programme

Study population	Cleaners	Health care workers	Construction workers	Industrial workers
**Predominant sex**	Females	Females	Males	Males
**Physical workdemands**	Repetitive work	Heavy lifting	Heavy lifting	Repetitive work
	Moderate force demands	High force demands	High force demands	Moderate force demands
**Individual capacities and health profile**	Low physical capacities	Low physical capacities	Low physical capacities	Low physical capacities
	Musculoskeletal disorders	High prevalence overweight	High prevalence overweight	Musculoskeletal disorders
**Interventions**				
Strength Exercise		**X**	**X**	**X**
Coordination Exercise	**X**			
Aerobic Exercise		**X**	**X**	**X**
Cognitive Behavioral Training	**X**	**X**		**X**
Participatory Ergonomics				**X**
Diet		**X**		

### Study areas

The FINALE programme is conducted in different regions of Denmark. In specific, the industrial workers are recruited from a single large workplace in the Southern region. The health care workers are recruited from public health-care centres in a county of Jutland. The cleaners are recruited from several large work-places (both private and public) situated in Zealand employing at least 30 cleaners. The construction workers are primarily working in the Southern region.

### Conceptual model

**Figure 1:**
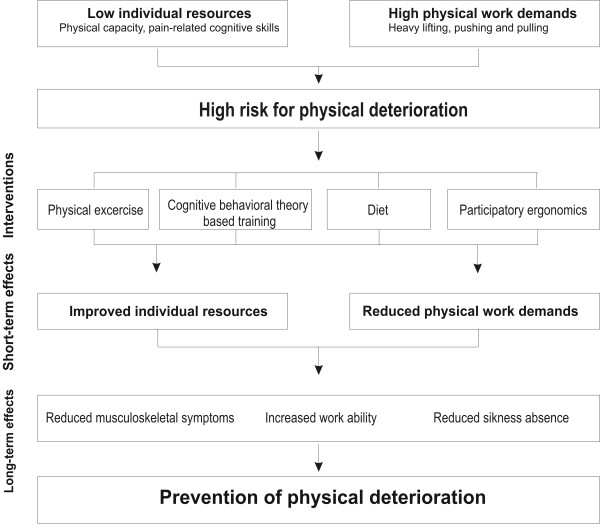
**The conceptual model of the FINALE programme**.

The conceptual model of the FINALE programme is illustrated in figure 1. The selected job groups are generally characterized by a mismatch between the individual physical capacities and physical work demands, providing the high prevalence of physical deterioration. Because of the different physical capacities, physical work demands and health profile of the selected job groups, the initiatives for preventing physical deterioration need to be specifically tailored for each job group. The initiatives for preventing physical deterioration in the FINALE programme are physical exercise training, participatory ergonomics, diet and CBTr. The initiatives are described in more detail in the next section (i.e. main initiatives of the FINALE programme). Common for all initiatives is that they are aiming at balancing the mismatch between the physical capacities and physical work demands for preventing physical deterioration. All initiatives are considered to impose rather acute effects (within 3 months) on the balance between the individual capacities (i.e. muscular strength, aerobic capacity, postural stability and kinesiophobia) and the physical work demands (i.e. physical exertion and reduced excessive body weight), resulting in reduced physical deterioration (i.e. reduced musculoskeletal disorders, improved work ability, and reduced sickness absence) in the longer perspective (1 year).

### Main initiatives of the FINALE programme

#### Physical exercise training

A novel approach of the FINALE programme is that the type of physical exercise training is tailored to the physical exposure at work, physical capacities and health profile of each respective job group. For decades, high physical capacities have been considered important for preserving work ability among workers with high physical demands [54], and workers with low muscular strength of the trunk and neck/shoulder region are considered to have an impaired tolerance for physically heavy work [45,55]. Hence, low muscular strength is a predictor for work disability in home care workers [12] and low endurance of back muscles is a predictor for first time occurrence of low back pain in men [56]. Because high physical work demands do not have the same positive effects on physical capacities as leisure time physical activity [21-25], the need for physical exercise training for maintaining or improving physical capacities may be particularly important for workers with high physical work demands. Accordingly, participation in leisure time physical activity is shown to be particularly important for reducing the relatively high risk for premature cardiovascular and all-cause mortality among men with moderate and high physical work demands [16]. It is likely that such physical exercise training need to be carefully planned regarding type, frequency and intensity for having positive effects on workers with physically heavy jobs. Among employees in sedentary work, introducing a wide range of physical activities is shown effective for improving health and preventing musculoskeletal disorders [39,57]. However, among employees with high physical work demands, the optimal strategy may rather be to increase the physical capacity necessary for performing the physical work demands. Therefore, the interventions in FINALE are tailored to the specific physical demands of the employees in the respective job group.

#### Cognitive behavioral theory based training (CBTr)

Psychosocial factors are well accepted to have an important role for perception, experience and behaviours regarding musculoskeletal disorders [58,59]. Because consequences of pain are influenced by the pain tolerance [60-63], a bio-psychosocial frame of reference is recommended for rehabilitation of chronic pain patients [64]. The general objective of cognitive behavioral therapy interventions is to identify dysfunctional attitudes and coping behaviours, suggest functional alternatives, and train and implement these in every day life [65,66]. There is evidence that cognitive behavioral therapy interventions are effective for reducing chronic pain and pain related behaviours [66-68] and facilitate return to work [31,32]. Therefore, it is considered the preferred psychological treatment for patients with pain-related disability [64]. Moreover, cognitive behavioral therapy interventions focusing on risk factors for prolonged pain and disability (e.g. fear of pain and movement) are shown effective for reducing days of sickness absence and return to work [69,70], and critical for rehabilitation of work disability [63,71]. However, the effects of cognitive behavioral therapy interventions for primary prevention of musculoskeletal disorders, reduced work ability and sickness absence among working employees have not been previously investigated. Therefore, a CBTr method was developed and tailored to a preventing group based protocol, aiming at employees remaining at work

#### Participatory ergonomics

Participatory ergonomic initiatives involve active participation and cooperation between the workers and management in the process of recognizing risk factors in the workplace, and to choose the best solution to reduce the risks [72]. Participative ergonomic initiatives are reported to be effective for preventing musculoskeletal disorders [73-75] and sickness absence [72,73,76].

However, the effectiveness of ergonomic interventions in general has been questioned [77]. This may be due to participatory ergonomics not necessarily being advantageous for all job groups. For example, participatory ergonomic initiatives may be particularly effective in job groups with high physical work demands, repetitive movements and high levels of musculoskeletal disorders, like sewers and garment workers [78,79]. In such job groups with high physical work demands and repetitive work, improved ergonomic work conditions have the potential to provide a significant reduction in rate of physical exertion at work [80].

#### Diet

Obesity is a considerable public health problem, being associated with chronic diseases like type 2 diabetes, cardiovascular disease and mortality [81-83]. In the working population, obesity is shown associated with increased risk for musculoskeletal disorders [84], sickness absence [85], and work disability due to osteoarthritis and cardiovascular disease [29]. Diet alone has shown limited effectiveness for long term weight loss [86]. This is considered to be due to dietary constrictions reducing the energy metabolism [87], making it more difficult to achieve and maintain long term weight loss. Combining physical exercise training (enhancing the energy expenditure) with diet is therefore recommended for long term reductions in body weight [88]. However, programmes combining diet and exercise training are shown insufficient for long term weight loss [86]. CBTr is therefore recommended to be combined with diet and physical exercise training for supporting a healthy lifestyle, maintaining long term weight loss [89,90].

### Data collection and study materials

#### Self-reported measures

A structured self-administered questionnaire with validated measures (the FINALE questionnaire) was applied in all intervention studies. The questionnaire involved sociodemographic measures (e.g. age, sex, height, weight, ethnicity, country of birth, occupational group, employment status and education), lifestyle behaviour and health (e.g. smoking, alcohol consumption, medicine use, sleeping behavior, the International Physical Activity Questionnaire [57], General health and Vitality, SF-36 [91,92], Standardized Nordic Questionnaires for the analysis of musculoskeletal disorders [93], sickness absence [94], general self-efficacy [95], Tampa Scale of Kinesiophobia [96], work-related factors (e.g. exposure to specific physical work tasks) [97], Copenhagen Psychosocial Questionnaire [98], Work ability Scale and Index including chronic diseases and sickness absence [99,100], Work Limitation Questionnaire [101], and perceived physical exertion [102].

A short message service (SMS) based method was applied in two of the RCT (cleaners and construction workers). Each week, the participants received 4 questions on their mobile phone during the entire intervention period. The participants received the questions on Wednesday, with a reminder on Sunday. The 4 questions concerned 1) neck-shoulder pain intensity last 7 days (scale 0-9), 2) low-back pain intensity the last 7 days (scale 0-9), 3) work ability the last 7 days (scale 0-9), and 4) hours of leisure time physical activity the last 7 days. The questions were inspired from the Standardized Nordic Questionnaires for the analysis of musculoskeletal disorders [93], the Work ability Index [99,100] and the International Physical Activity Questionnaire [57].

#### Objective measures

The objective measures of the RCTs are height, body weight, hip/waist-ratio, percentage body fat with impedance recording, blood pressure, muscular strength of neck, shoulder, trunk flexion and extension [103], postural stability [104] and aerobic capacity [105] with some variation between intervention studies on the number of objective measures. Sickness absence data are retrieved from the workplaces.

#### Recruitment and randomization of participants

In all 3 RCTs, the recruitment and randomization of participants were done in according to the same principles. For each respective RCT, the participants were recruited from managers' lists of employees, which included their civil registration number with linked information about age and sex. All eligible employees were invited to an information meeting during their working hours and asked to fill out a screening questionnaire and to give consent or not to enrol in the study.

For minimising contamination, a restricted cluster randomisation was applied for allocating the employees into the intervention and reference groups. Each workplace or worksite was considered a stratum. Clusters depended on work teams - where possible - were made up from groups in which employees had lunch, worked in close proximity to each other, or worked to the same manager. Clusters were matched on sex, age, job seniority or job type with varied block sizes. The randomization process was concealed, and performed after the baseline measures, and those assessing the outcomes were blinded to group assignment.

### Outcomes

The common main outcomes of the interventions composing the FINALE programme are the measures for physical deterioration; musculoskeletal disorders, work ability and sickness absence. However, the primary and secondary outcomes are specifically tailored for each respective intervention.

### Description of the intervention of the FINALE programme

#### Intervention among cleaners (The FINALE - Clean study)

The title of this study is "Preventing deterioration among cleaners". The project is ethically approved by The Committees on Biomedical Research Ethics of the Capital Region of Denmark on the 13th May 2008 (ref: H-C-2007-0033) and qualified for registration in the International Standard Randomised Controlled Trial Number Registry (ISRCTN96241850). The main aim of the study is to evaluate the effect of interventions with physical exercise training and CBTr among cleaners on musculoskeletal disorders, physical capacity, kinesiophobia, work ability and sickness absence.

The participants are cleaners working at least 20 hours per week. The intervention is a 1 year RCT, taking place during the working hours of the cleaners. The previously described self-reported and objective measures are obtained before the intervention period, after the first phase (3 months) and after the second phase (12 months). Exclusion criteria to the project are pregnancy, angina pectoris and life-threatening diseases according to safety regulations for physical evaluation methods [103]. Primary outcome measures are work ability and sickness absence from the participating companies' annual registrations. Secondary outcomes are musculoskeletal disorders, physical capacity (muscular strength and postural stability) and kinesiophobia. The data will be analyzed by ANOVA models on an intention to treat basis.

**Figure 2:**
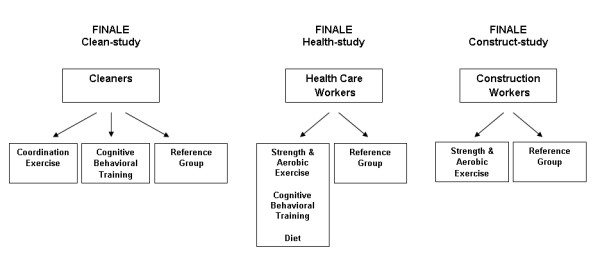
**The 3 randomized controlled trials of the FINALE programme with underlying arms and interventions offered**.

The cleaners are randomized into I) Physical exercise training (physical coordination training), II) CBTr or III) Reference group (Figure 2). I) The physical coordination training is divided into 2 phases. In the first phase comprising the first three months of intervention, weekly sessions of 20 minutes' duration at the workplace are performed with guidance from an instructor. The physical exercise training consists of intensive physical coordination training, providing high activation of stabilising muscles around the trunk and shoulder girdle. This intensive physical coordination training is designed for improving muscular strength and postural stability of the cleaners. In the second phase comprising the following 9 months, the number of training sessions is gradually reduced, with only 1 session per month the last 6 months. In this phase, the participants are introduced to several new types of physical exercise training based on the participants' preferences. II) The CBTr occurs in groups guided by a CBTr trained group leader, and is also divided in 2 phases. The first phase comprising the first 3 months of intervention, consists of 2 monthly sessions at the work place of 2 hours duration. The CBTr mainly comprises group discussions of issues regarding pain-related dysfunctional attitudes like kinesiophobia, coping and management, with facilitation of functional alternatives. Moreover, the CBTr also involves education of physical activity, problem solving, applied relaxation techniques and practice of the coping skills in their home environment [32]. In the second phase comprising the following 9 months, the number of training sessions is gradually reduced, with only 1 session of 1 hour duration per month the last 6 months. In this phase, the experiences and considerations of the cognitive and behavioral changes of the participants from the first phase are debated, and reflections and support for how to obtain long-lasting cognitive and behavioral changes are in focus. III) The reference group receives a health check of 1 hours' duration, including a pulmonary-function test and aerobic capacity test [105]. The intention of the health check is to give the participants some output for their participation in test and questionnaire sessions.

#### Intervention among health care workers (The FINALE - Health study)

The title of this study is "Physical exercise, dietary counseling and cognitive behavioral training as a combined intervention to reduce weight and to increase workability in health care workers". The project is ethically approved by The Central Denmark Region Committees on Biomedical Research Ethics on the 7th may 2009 (ref: M-20090050), and qualified for registration in the International Standard Randomised Controlled Trial Number Registry (NCT01015716). The main aim of this study is to evaluate the effect of a 1-year lifestyle intervention aiming at reducing body weight, increasing work ability and reducing sickness absence among health care workers.

The participants are health care workers employed at the municipality of Randers in Denmark. The intervention is a 1 year RCT, mainly taking place during working hours, but also with some physical exercise training during leisure time. The previously described self-reported and objective measures are obtained before the intervention period, after the first phase (3 months) and after the second phase (12 months).

Exclusion criteria to the project are pregnancy, angina pectoris and life-threatening diseases according to safety regulations for physical evaluation methods [103]. The primary outcome measure of this study is measured body weight. Secondary outcomes are work ability, musculoskeletal disorders, sickness absence, physical capacity (aerobic capacity and muscular strength) and fat free mass. The data will be analyzed by ANOVA models on an intention to treat basis. The participants are randomized into: I) intervention group; offered weight loss, physical exercise training and CBTr, II) reference group; offered monthly group seminars on health related topics (Figure 2). The intervention is divided in 2 phases with 60 min weekly sessions through-out the year in working hours. The first phase of 3 month focuses mainly on diet changes and weight loss and each participant is given a personal diet calculated to give an approximately 1000-1200 kcal/day energy deficit to achieve weight loss. Body weight and fat mass is monitored in weekly sessions. During this phase physical exercises (both strength and aerobic training) are introduced at the weekly sessions and CBTr is given mainly with focus on diet change. During the second phase lasting 9 months, weekly sessions are continued with focus placed on exercise. Exercises are performed to achieve improved aerobic capacity as well as strengthening exercises during the work hour sessions. In addition, exercise is planned for leisure time for further 2 hours each week. Exercise diary is kept throughout the intervention. During the second phase, dietary counselling is aimed at achieving weight maintenance and following national nutritional recommendations. CBTr during this phase focuses on changes in exercise habits and weight maintenance. The reference group receives invitation to attend a monthly seminar of 2 hour duration on a wide range of health related topics, but no practical guidance is given.

#### Intervention among construction workers (The FINALE - Construct study)

The title of this study is "Effects of specified work site physical activity intervention on musculoskeletal disorders among employees with physically heavy work". The study protocol is approved by the Ethical Committee in region Southern Denmark (S-20090058), and qualified for registration in Clinical Trials.gov (NCT01007669). The aim of the study is twofold:

1. to outline the extent of musculoskeletal disorders in workers with heavy work load in relation to physical and metabolic fitness through a health check, which includes also assessment of general physical activity and working condition.

2. to evaluate the effect of individually adjusted exercise programs on muscular strength, aerobic capacity, musculoskeletal disorders, work ability and sickness absence.

The participants are construction workers working at least 20 hours per day. The project is a RCT study with two arms (Figure 2). Primary effect variable is muscular strength (back and trunk) and aerobic capacity. Before and after the intervention period, all participants complete a health check. The health check constitutes individual health profiles and includes measures of anthropometry, blood pressure, aerobic capacity, muscular strength, percent body fat, and blood lipids. In connection with the health check, the participants wear an Actiheart for 7 days to measure physical activity at leisure and at work. All participants fill out the FINALE questionnaire both at first and the second health check, and after one year (follow up). Results from the first health check form the basis for the design of the specific type of exercise intervention offered to the intervention group for 3 months.

The intervention group accomplishes supervised physical exercise training 3 times a week for 20 minutes. The physical exercise training takes place during the working hours of the construction workers. The contents of the physical exercise training are aerobic and muscular strengthening training. The aerobic training program is cycling on a bicycle ergometer with intensity corresponding to minimum 70% of maximal heart rate, and the muscular strength training program consists of 3 standardized exercises for the body regions: shoulder girdle, trunk/back and lower extremities. The intensity of the muscular strength training aims at 15RM. The training program is individualized regarding the relative duration of aerobic and muscular strength training as well as the number and specific choice of strength exercises based on the health profiles. The reference group receives, in addition to health check and physical activity measurements, one 2 hours informational meeting where the topic is healthy lifestyle.

#### Intervention among industrial workers (The FINALE - Indust study)

The aim of this case-control study was to investigate effects of a participatory ergonomic intervention on musculoskeletal disorders, work ability, kinesiophobia and sickness absence.

The participants are industrial workers from a large company. The intervention is regarded as an exploratory study, being evaluated in a matched case-control design. Questionnaire surveys of all employees at the company were performed before and after the intervention period. According to Danish law, questionnaire and register based studies do not need approval by ethical and scientific committees, nor informed consent. Since this research project only involved questionnaires, the study has been notified to and registered by The Danish Data Protection Agency. The primary outcome variable is rate of physical exertion during work, with musculoskeletal disorders and kinesiophobia as secondary outcomes.

The intervention is a company initiated offer to employees with pronounced musculoskeletal disorders, and conducted as a course among employees who registered, and were accepted as eligible after a clinical examination. The clinical examination was performed by the physiotherapist also conducting the intervention and being paid by the company. The company initiated the study because of high prevalence of musculoskeletal disorders and sickness absence among their non-administrative employees. The main principle of the intervention is that the degree of physically heavy work can be reduced, based on applied biomechanical knowledge. Secondly, specific training can make the employees take better care of their health in relation to the physical work demands, and finally learning to cope with pain and consider it as a friendly warning. The intervention was conducted in groups, and consisted of about 50 hours at the workplace and at a fitness centre during 4 to 5 months. The company paid all expenses for the course and the fitness centre, and the employees participated in their leisure time. All participants were video filmed during their normal work tasks, and this constituted the main material for the theoretical part. The theoretical part was supported by work tailored postural coordination exercises with guidance from an instructor. A matched control group was drawn from the employees not receiving the intervention, being matched on age, sex, musculoskeletal disorders and perceived physical work exposure based on the questionnaire survey performed among all employees before the intervention started. The researchers performing the matching procedure were not involved in selection of cases or conduction of the intervention.

### Statistical analysis

#### Power calculations

The power analysis in the FINALE - Clean study was based on work ability with a minimal relevant difference of 10% in a paired design and a power of 0.8 and a statistical significance level of 0.05. Mean and SD was based on interventions studies of work ability. To demonstrate a difference between the groups, 100 employees in each group are required. With an expected 20% drop-out, 120 participants are needed per group, and it is therefore intended to recruit 360 cleaners to the study.

The power analysis in the FINALE - Health study was based on a minimal relevant difference of 3 Kg difference in body weight and a power of 0.8 and a statistical significance level of 0.05. Mean and SD was based on interventions studies of weight reductions. To demonstrate a difference between the groups, 30 employees in each group are required. With an expected 30% drop-out, 43 participants are needed per group, and it is therefore intended to recruit 86 health care workers to the study.

The power analysis in the FINALE - Construct study was based on a minimal relevant difference of 10% in muscular strength and aerobic capacity ( O_2max_) and a power of 0.8 and a statistical significance level of 0.05. Mean and SD was based on interventions studies of muscle strength in neck/shoulder [39]. To demonstrate a difference between the groups, 46 participants in each group are required. With an expected 20% drop out, 60 participants are needed per group, and it is therefore intended to recruit 120 construction workers to the study.

In all respective RCTs, evaluation of intervention effects on the primary outcomes from the baseline measurement to the 3^rd ^and 12^th ^month follow-up measures, intention-to-treat analysis with subsequent per-protocol completers analyses will be conducted. The large amount of common objective measures and questionnaire variables from the 3 RCTs will be merged into a common data base. This will allow for cross-analysis performed over the 3 RCTs to evaluate effective components of the interventions as well as the overall effect of the tailored intervention concept on work ability among employees with physically heavy work

## Discussion

Physical work demands exceeding the safety margin of the individual physical capacities (i.e. poor work ability) is generally considered to enhance the risk for physical deterioration, defined as musculoskeletal disorders, poor work ability and sickness absence. However, effective interventions for preventing physical deterioration in job groups at high risk remain to be established. The aim of the FINALE programme, being an umbrella for 4 tailored interventions among job groups with high risk for physical deterioration, is to evaluate the effects of balancing the relation between individual capacities and physical work demands on physical deterioration. The background, design, conceptual model and interventions of the FINALE programme have been described.

### Strengths and limitations of the FINALE programme

A strength of the FINALE programme is that it constitutes 3 RCTs tailored to the physical work demands, physical capacities and health profile of different job groups characterized by a high risk for physical deterioration. This feature of the FINALE programme enhances the probability for enabling evidence-based information for public health policy and health promotion strategies among employees in job groups with high risk for physical deterioration. Another strength of the FINALE programme is that all interventions take place at workplaces, providing a high external validity of the findings. An additional strength is that several workplaces with different characteristics (e.g. rural, urban, private and municipal) from different regions of Denmark are included in the FINALE programme. Moreover, numerous subjective and objective work and health-related measures are collected. Because the same FINALE-questionnaire and many common objective measures are included in the studies, the data from all interventions can potentially be merged and analysed.

A limitation of the FINALE programme is that only simple measures of process evaluation such as proportion of workers in uptake, actual start of the programme and actual completion of the RCT are collected. Moreover, no economical cost-effectiveness evaluations are included. Another limitation is that the intervention among industrial workers is an exploratory, not well controlled study.

### Impact of results

The study population of the FINALE programme (i.e. employees in job groups with high physical demands) is well documented to have a high risk for physical deterioration. If proven effective, the specific tailored interventions to the different job groups can provide meaningful scientifically based information for public health policy and health promotion strategies for employees in these job groups at high risk for physical deterioration. This knowledge can be beneficial for occupational health professionals, supervisors, companies and employees in these job groups. Because the interventions are carried out during ordinary circumstances at a wide range of Danish workplaces, it is expected that the findings can be transferred and interventions implemented in other workplaces with high physical demands.

## Competing interests

The authors declare that they have no competing interests.

## Authors' contributions

AH led the writing of the manuscript, and wrote the first draft of the abstract, background, methods and discussion. All authors contributed to the design and protocol of parts of the FINALE programme. KS is responsible for conception and design of the FINALE study and wrote the grant application, with contributions from MBJ, JRC, GS, JEA. MBJ and KS wrote the specific information about the FINALE - Clean study. JRC and KO wrote the specific information about the FINALE - Health study. BG and GS wrote the specific information about the FINALE - Construct study. AH and KS wrote the specific information about the FINALE - Indust study. All authors read and approved the manuscript.

## Pre-publication history

The pre-publication history for this paper can be accessed here:

http://www.biomedcentral.com/1471-2458/10/120/prepub
